# Revisiting the Role of Individual Variability in Population Persistence and Stability

**DOI:** 10.1371/journal.pone.0070576

**Published:** 2013-08-02

**Authors:** Andrew Morozov, Anna F. Pasternak, Elena G. Arashkevich

**Affiliations:** 1 Department of Mathematics, University of Leicester, Leicester, Leicestershire, United Kingdom; 2 Laboratory of Plankton Ecology, Shirshov Institute of Oceanology, Moscow, Russia; University of Leeds, United Kingdom

## Abstract

Populations often exhibit a pronounced degree of individual variability and this can be important when constructing ecological models. In this paper, we revisit the role of inter-individual variability in population persistence and stability under predation pressure. As a case study, we consider interactions between a structured population of zooplankton grazers and their predators. Unlike previous structured population models, which only consider variability of individuals according to the age or body size, we focus on physiological and behavioural structuring. We first experimentally demonstrate a high degree of variation of individual consumption rates in three dominant species of herbivorous copepods (*Calanus finmarchicus*, *Calanus glacialis*, *Calanus euxinus*) and show that this disparity implies a pronounced variation in the consumption capacities of individuals. Then we construct a parsimonious predator-prey model which takes into account the intra-population variability of prey individuals according to behavioural traits: effectively, each organism has a ‘personality’ of its own. Our modelling results show that structuring of prey according to their growth rate and vulnerability to predation can dampen predator-prey cycles and enhance persistence of a species, even if the resource stock for prey is unlimited. The main mechanism of efficient top-down regulation is shown to work by letting the prey population become dominated by less vulnerable individuals when predator densities are high, while the trait distribution recovers when the predator densities are low.

## Introduction

Mathematical models of population dynamics have been actively implemented in theoretical ecology for almost a century and yet our models often fail to adequately mimic the complex ecological patterns observed in nature. One way to make ecological models more realistic is taking into account the fact that within each population individuals are rarely identical. Rather, there often exists a pronounced degree of variability of organisms; in terms of traits such as the developmental stage, age, body size, accumulated energy, physiological life traits, individual behaviour, etc [1]–[6]. This fact is now well recognized in theoretical ecology and there exists a large amount of publications on modelling dynamics of such structured populations using various frameworks [4], [7]–[11].

Most of the existing models of structured populations, however, consider the situation where intra-population variability is due to differences in age or in body size, while there is a growing body of evidence that suggests that individuals within a population largely vary according to other characteristics which are not related to the age or the body size. That is, organisms of the same developmental stage usually differ regarding behavioural aspects or physiological life traits (e.g. the ability to disperse, consume food, vulnerability to predation, etc). In particular, a large number of experimental data reveal a high degree of intra-population variability in terms of the behavioural strategies that a particular individual uses to optimise its fitness [2], [5], [6], [12]–[14]. To be able to model such physiologically and/or behaviourally structured populations we cannot rely on the previous age-structured or size-structured models, where in the course of time each organism gradually evolves from a lower to a higher cohort [4], [7], [9]-[11]. Organisms are known to be capable of keeping their personalities over their entire lifespan [2], [6], [15], [16] and this requires a different framework for the description of such systems [17]. The term ‘physiologically structured populations’ is still often understood in the modelling literature to mean structuring in terms of body size or developmental stage - or sometimes considering the feeding history- [4], [7], [18], thus neglecting other possibilities of structuring such as the choice of feeding strategy (but see [17], [19], [20]).

To partially fill this gap, here we suggest a parsimonious model where a population is structured according to physiological or behavioural properties, and investigate how such inter-individual variability would affect the persistence of the whole population under variable predation pressure. Namely, we consider a predator-prey model (based on an integro-differential setup), where the prey population is structured according to their growth rate and vulnerability to predation. Unlike previous models, we consider that the life history traits of a given individual do not change across the whole life span; however, offspring can acquire different life history traits than their parents.

The main goal of this paper is revisiting the role of intra-population structuring of the prey population in the persistence and stability of predator-prey systems. As an important case study, we model trophic interactions between herbivorous and carnivorous zooplankton or planktivorous fish in the ocean. It is well known that zooplankters of the same developmental stage show a large individual variability, including variation in swimming and feeding rates, [1], [21], [22] and this can be related to intra-population difference in the foraging behaviour of grazers (see section 2 for more detail). We consider persistence of populations in eutrophic ecosystems, where competition between grazers for resources can be neglected and the major factor controlling herbivores is top-down control by their predators [23],[24].

Based on a series of feeding experiments in the laboratory, we demonstrate that individual zooplankton grazers (marine copepods) exhibit a pronounced disparity in their food consumption rate. The large intra-population variability in feeding rates observed in laboratory feeding cannot solely be explained by random variation in the ingestion of individuals in each experiment; rather, this variation is intrinsic and signifies the existence of a permanent structuring of grazers according to their consumption capacity. We then implement the new predator-prey model which considers intra-population variability of zooplankton and show that structuring of grazers ought to enhance the population persistence, thus preventing the species’ extinction. Interestingly, stabilization of plankton communities becomes possible for an infinite carrying capacity of the system (i.e. an unlimited stock for the grazers). We show that the stabilization and persistence in the model is rather robust with respect to different types of trade-off relations between the feeding rate and vulnerability to predation, making our results applicable for some other, non-planktonic, ecosystems.

## Methods

### Revealing Intra-population Variability of Zooplankton in Feeding Experiments

In this section, we experimentally demonstrate a large degree of intra-population variability in the zooplankton food intake rate. We have investigated the feeding of three species of herbivorous copepods: *Calanus glacialis* (collected in the vicinity of Svalbard); *Calanus finmarchicus* (Norwegian fjords near Tromsø) and *Calanus euxinus* (Northern Black Sea). To avoid any influence of the body size or the age of grazers in experiments we compared specimens of the same copepodite stage: stage CV for *C. glacialis*; stage CVI (females) for *C*. *finmarchicus* and CV for *C. euxinus*. The technical details related to experimental estimations of the ingestion rates of zooplankton grazers are given in the Material S1.

**Figure 1:**
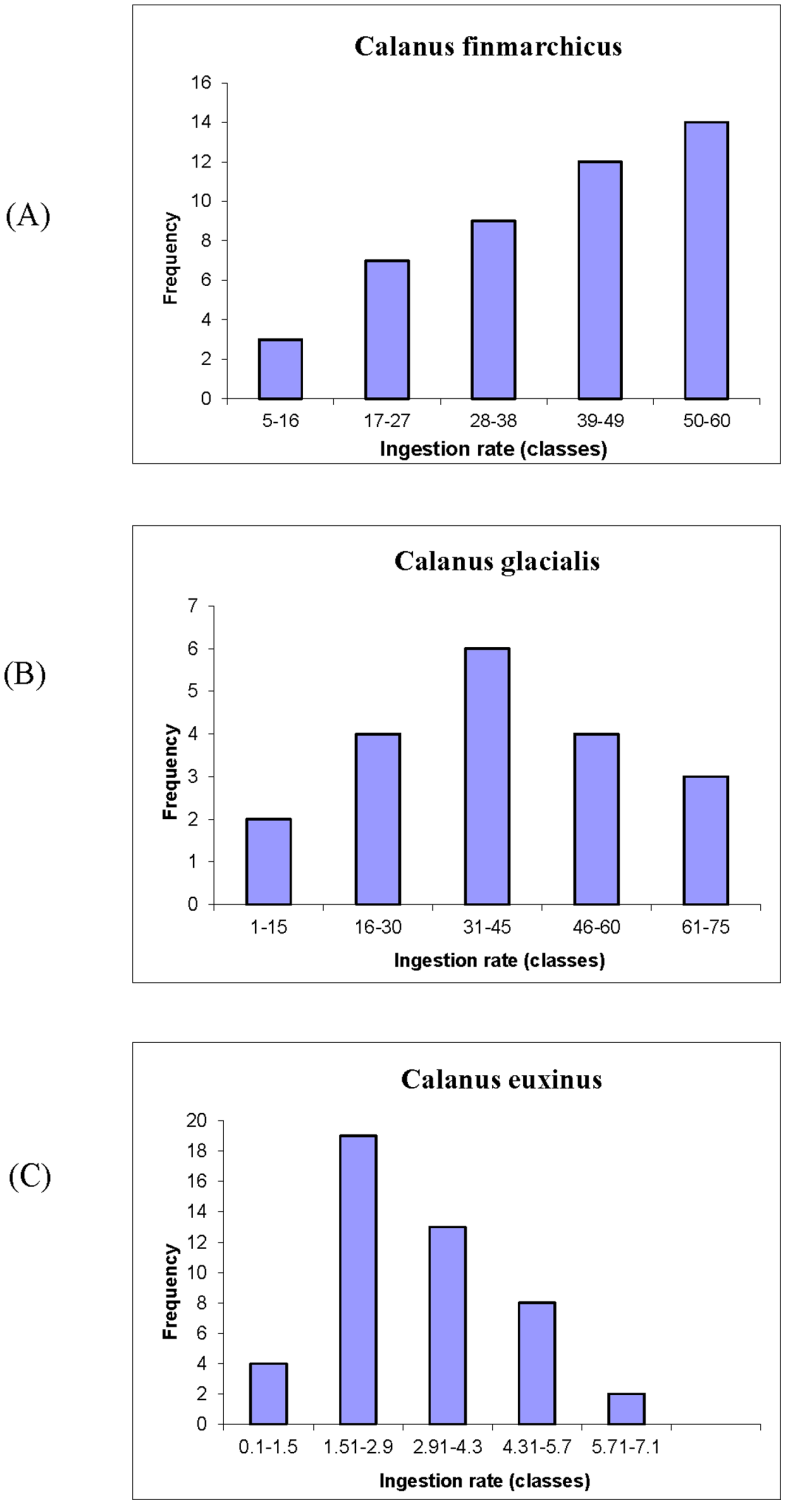
Individual ingestion rates of the three different *Calanus.* species measured in the laboratory. The number of feeding sessions is 5, 6 and 6 for *C. finmarchicus*, *C. glacialis*, and *C. euxinus*, respectively. One can observe a pronounced variation of ingestion rates, which can be estimated by computing the coefficient of variation defined as *CV* =  *σ/m* (in all cases the calculation gives *CV* ≈0.5).

**Figure 2:**
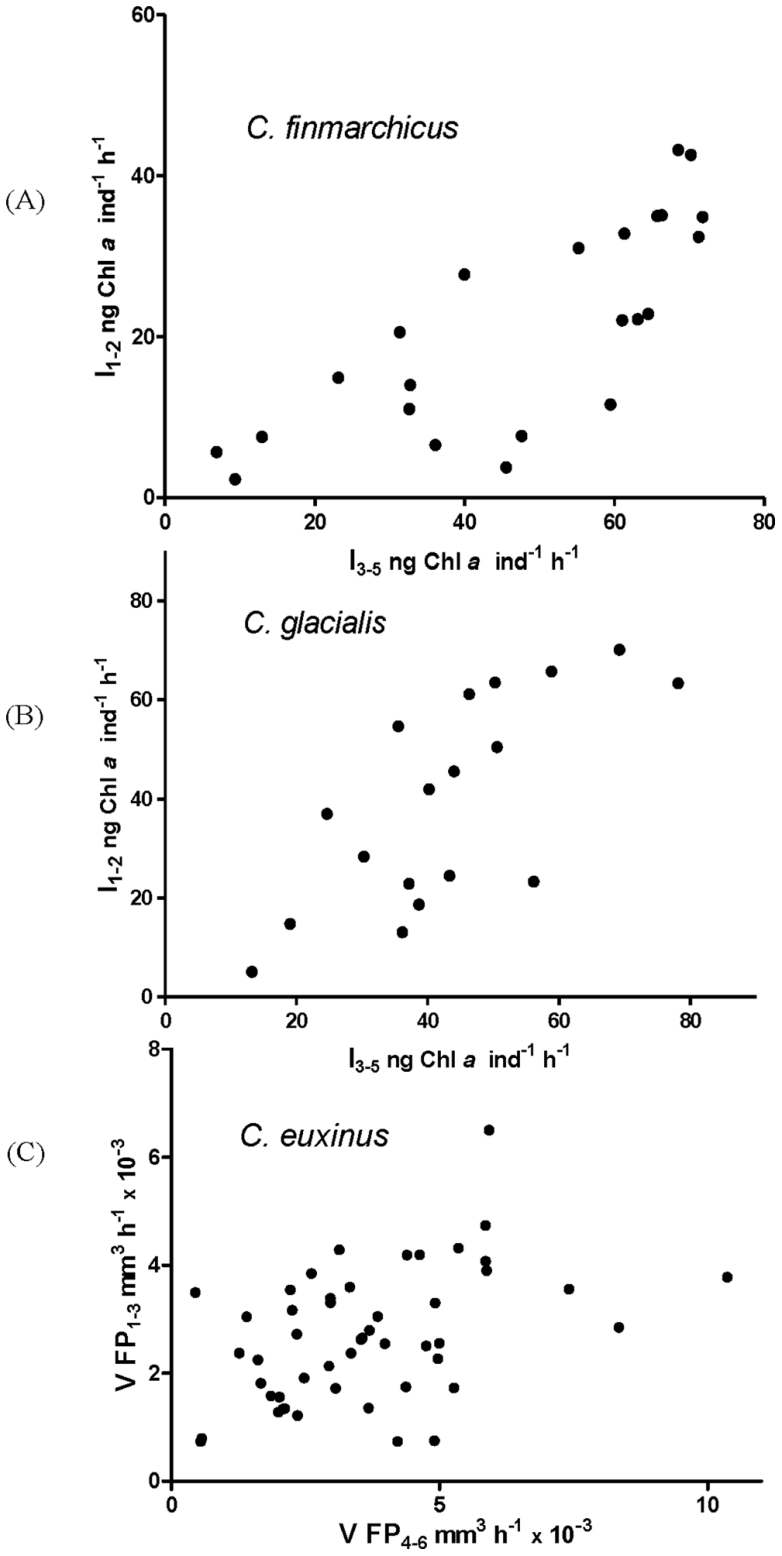
Separating the ‘true’ physiological structuring in the population of grazers from possible effects of random variation in different experiments. For each specimen the ingestion rate averaged over the first part of experiments is plotted against the ingestion rate averaged over the second part of experiment. The estimated correlation coefficients are shown in each graph. In all cases positive correlation (determined by taking Spearman correlation coefficients) between the two parts of experiment was found to be statistically significant, the correlation coefficients being, respectively, *R* = 0.82; *R* = 0.72; *R* = 0.54 (*P*<0.0001).

Overall, investigation of inter-individual variation was completed using 25 specimens of *C. glacialis*, 25 specimens of *C. finmarchicus* and 50 specimens of *C. euxinus*. Five subsequent feeding sessions (1 session per day) were run for *C. glacialis* and *C. finmarchicus* and 6 sessions - for *C. euxinus*. The results of our experiments are shown in Figs.1, 2. In particular, Fig. 1 shows the ingestion rates averaged over the duration of the experiment (i.e. over all the feeding sessions). One can see from this figure that for all species the average consumption rate exhibits a pronounced variability, with such intra-population variability also being observed within the same feeding session (we do not present this result here). One can see that the ratio between the maximal and minimal ingestion rate of different individuals can be as large as 20–40 (*C. glacialis*), 40–70 (*C. finmarchicus*) and 40–70 (*C. euxinus*). The degree of variability of ingestion rates can be estimated more accurately, based on the standard deviation *σ* ^2^, showing scattering of the data around the estimated “population” mean, *m*. We obtained that *m* = 41.0, *σ* = 17.3 (*C. glacialis*), *m* = 38.3, *σ* = 17.7 (*C. finmarchicus*) and *m* = 9.7, *σ* = 5.0 (*C. euxinus*). One can see that the ratio between *σ* and *m* (called the coefficient of variation) for each case is rather close to 50% which indicates a large degree of variability in the feeding rates.

We further checked whether a high/low/intermediate level of the intake rate was individually specific- that is, whether a certain level of feeding activity was typical for a given specimen- in order to check whether the pronounced variability was observed only due to random variation of individual feeding rates, with the mean feeding rate of different individuals actually being close to each other. Indeed, one can suggest that specimen *i* feeding at a high rate during feeding session 1 could drop feeding in session 2, and specimen *j* can show the opposite behavior, etc. The results obtained, however, demonstrated that specimens of all three species were maintaining their specific individual feeding rate, as can be seen from Fig. 2. For each individual we plotted the ingestion rate averaged over the first half of the feeding sessions against the rates averaged over the second half of the feeding sessions. We have estimated the correlations between the two ingestion rates. One can see that there is a strong link between the two ingestion rates since there is highly significant correlation which clearly indicates that the difference in ingestion rates of individuals cannot be explained by random variation of consumption rates for a given specimen in different experiments. The same conclusion can be made using another partition of sessions (e.g. comparing sessions 1,3,5 with sessions 2,4,6). Finally, for each species we have checked if the feeding conditions for the whole population can change, for instance, because of adaptation to food or some other reasons. The results obtained showed that specimens of all three species maintained their individual-specific feeding rates (one-way ANOVA test, *p*<0.0001). Interestingly, the variation observed cannot be explained by the difference in the size of animals, since in each experiment the organisms were chosen from a given stage and species and therefore had similar body size. Also, we have not found any statistically significant correlation between the sizes of animals and their food consumption rate.

The above experiments allow us to make an important conclusion: zooplankton individuals may exhibit a pronounced disparity of feeding rates which is related to physiological/behavioral particularities of each individual, and not related to their body size or age (i.e. copepodite stage).

On the other hand, it is known that variation in zooplankton feeding rate as well as in swimming ability can be related to intra-population difference in the growth rate [1], [21], [22]. It was well reported that as a part of the optimal foraging behaviour zooplankton often migrate to the surface layer of the ocean with higher food abundance, in spite of the increased predation risk [25]–[27]. The grazers often use the so called ‘eat and run’ strategy, which consists in quickly filling the gut and immediately leaving the risky environment [27], [28]. The intrinsic ability of filling the gut can substantially vary from individual to individual [1], [29]; due to variation in concentration, activity or the rate of production of digestive ferments. Additionally, individuals with high ingestion rates can leave the surface layers faster than the others, thus spending less time inside the risky environment [30], [31]. Thus, variation in physiological traits within a zooplankton population can translate itself into different foraging strategies, thus resulting in difference in the growth rates of individuals.

### Modelling Framework

To describe trophic interactions between zooplankton grazers and their predators we use a standard predator-prey model [32]. However, we introduce an integral term to take into account structuring of the prey population. The model equations read as follows
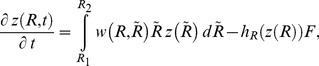
(1)

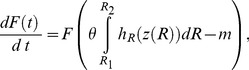
(2)where z(*R*) is the function describing the distribution of zooplankton individuals across the cohorts according to a certain life history trait which is characterized by the parameter *R*. In this paper we consider *R* to be the per capita growth rate *R*; however, it can potentially be any other parameter (e.g. the feeding rate). The product *z*(*R*) *dR* approximately gives the biomass of zooplankton with the trait *R* varying between *R* and *R+dR*; the total biomass of zooplankton Z can be obtained by integration of *z*(*R*) from the minimal and maximal possible values of *R*, i.e. from *R_1_* to *R_2_*. *F* is the total biomass of predator; for the sake of simplicity we consider here that the predator population is unstructured. The parameters *θ* and *m* are the food utilization coefficient and the mortality rate of the predator, respectively.

The integral term in (1) describes the growth rate of zooplankton due to the reproduction of all cohorts. The weight 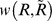
describes the contribution of cohort 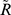
to the birth rate of individuals of cohort *R* and it has a meaning of a certain demographic kernel giving the distribution of demographic factors. We assume that the demographic factors are at genetic equilibrium, i.e. the demographic factors do not change in time (cf. [33]). For the sake of simplicity we consider that the per capita growth rate of each cohort is constant, i.e. we neglect the intraspecific competition in the zooplankton population; thus the carrying capacity of the system is infinitely large. We also require that integration of 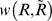 over all the cohorts should be equal to unity.
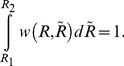
(3)


To proceed, one needs to specify the functional shape of the demographic weights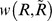. In this paper, we shall consider the following parameterization, which is a truncated normal (Gaussian) distribution of the per capita growth rate between the offspring
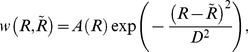
(4)where *D* is a positive parameter characterizing the half width of the above distributions; *A*(*R*) is the normalizing function which is needed to guarantee condition (3). Parameterization (4) takes into account the fact that the probability to have offspring with life history traits close to those of their parents is higher than probability to have some distinct traits. Note that numerical analysis shows that the use of similar parameterizations (e.g. parabolic parameterization) of 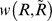 would give qualitatively similar bifurcation diagrams.

We consider that the functional response of the predator *h_R_* is of Holling type II given by the Monod parameterization [34].
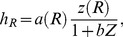
(5)where *b* is the parameter characterizing the saturation of predation at high prey density; *Z* is the total biomass of zooplankton. The coefficient *a*, which is proportional to the attack rate, is different for different zooplankton cohorts; this parameter has the meaning of the vulnerability to predation and we suggest that *a* is a function of *R*: *a = a*(*R*). The half saturation rate 1/*b* can also depend on *R*, but for the sake of simplicity we consider it to be constant here, as relaxing this assumption by allowing *b* to vary with *R* does not qualitatively alter the main results (see Section 5). We neglect the natural mortality of prey compared to the losses due to predation. We should emphasize that here we consider structuring of the zooplankton population with respect to growth rate and vulnerability to predation, and we do not explicitly consider structuring of the population in space.

Finally, we need to specify the dependence of *a* on *R*, i.e. the function *a =  a*(*R*). We postulate that such a dependence, trade-off, exists, because such variation in the intensity of the feeding rate or the swimming abilities of the zooplankton individuals can influence the time spent by each individual grazer in the surface layer, where the risk of visual predation is high [27]. Another possible explanation for the existence of such a trade-off is that the feeding activity of a grazer can provoke strong microcurrents -thus indicating the presence of the grazer- and also increase the contrast between the transparent animal and the coloured full gut, which may help predators to locate their prey [35],[36]. To overcome this difficulty, we consider several possible parameterizations of *a = a*(*R*). In particular, we investigate the following generic trade-off relations
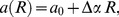
(6)

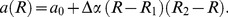
(7)


In the first case, the expression gives a monotonic relation between the growth rate and vulnerability to predation which can be either increasing (*Δα*>0) or decreasing (*Δα*<0). In the second case, the parameterization describes the situation where the dependence *a*(*R*) is a unimodal: for *Δα*>0 the predator attack rate is the highest for herbivores with intermediate growth rates. Alternatively one can consider the scenario where *Δα*<0 and the predators consume mostly herbivores with small or large growth rates, with the minimal attack rate for the prey in intermediate growth rates. Along with the simple parameterizations above, we have also briefly considered more sophisticated relations, in particular, those having several maxima and minima (see section 5 for a short discussion).

We consider a range of model parameters based on the literature [37]–[39]; however, we assumed broader limits for the parameters than in the cited papers, allowing for individual variation since the scattering of individual parameters can be rather large (e.g. Fig. 1). We considered that 0.025<*m*<0.1 1/day; 0.025<*θ*<0.5, 0<*a*<1 µg C l^−1^; 0<*b*<0.5 µg C l^−1^, 0.05<*R*<1.5 1/day. The unit of plankton density is chosen to be µg C l^−1^. Note that these values of parameters should be considered only as guidelines since model (1)–(2) is a generic model which does not pretend to provide high accuracy in terms of quantitative predictions. The other parameters (*D*, *Δα*, *a_0_*) were considered as control parameters and were varied within large ranges; however, we did require that 0<*a*<1 µg C l^−1^.

## Results

In this section we present an intensive investigation of model (1)–(2) including both analytical results and numerical simulations. We start with the simplest case, where there is no deviation from the parents’ foraging behaviour and so offspring inherit life history traits which are identical to those of their parents. In this case, the demographic kernel will be a delta function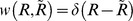, which we approximate in practice by choosing a very small *D* in (4), and the structured population becomes an ensemble of independent cohorts not related to each other (under the assumption that the carrying capacity is large enough and the individuals do not compete with each other for resources). Our simulations show that within a short time all but one cohort will quickly go extinct and system (1)–(2) becomes equivalent to a predator-prey model with the only prey cohort being the one with maximal value of *R* – *a*(*R*). It is well known that in such a system, the interior stationary state is globally unstable for a Holling type II functional response (with *b*>0) [40]. As a result, the stabilization of the system solely through top-down control is impossible. After a few oscillations with increasing amplitude the species density falls below a very low level, which would signify extinction of both species, thus only with some other factors can persistence of the species be ensured.

**Figure 3:**
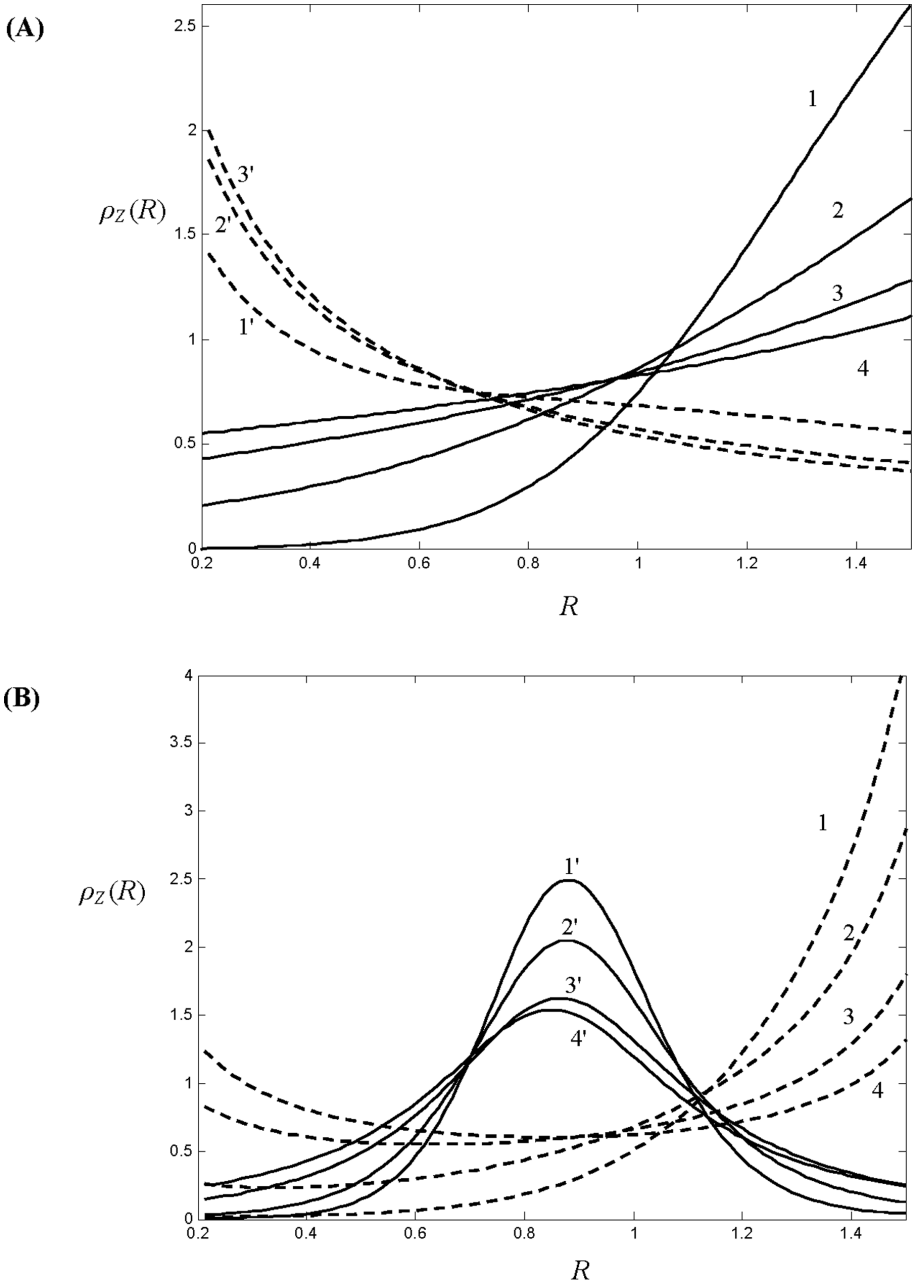
Density *ρ_z_* (*R*) of distribution of individuals in the prey population structured according to the growth rate *R* at equilibrium. (A) *ρ_z_* (*R*) are plotted for the linear trade-off relation *a*(*R*) given by (6). The solid line curves correspond to the case of a negative trade-off between *a* and *R* (*a_0_* = 1; *Δα = −*0.4); curves 1–4 are constructed for *D* = 0.35, 0.7, 1.5, 4, respectively. The dashed-line curves correspond to a positive correlation between *a* and *R* (*a_0_* = 0.2; *Δα = *0.72); curves 1′–3′ are constructed for *D* = 5, 1, 0.3. (B) *ρ_z_* (*R*) are plotted for the non-monotonic trade-off relation given by (7). The solid line curves 1′–4′ correspond to *Δα*<0 (*a_0_* = 1; *Δα = *−2), constructed for *D* = 0.2, 0.4, 1, 5, respectively; the dashed-line curves 1–4 correspond to *Δα*>0 (*a_0_* = 1; *Δα = *2) and plotted for *D* = 0.3, 0.45,1.2, 5. The other model parameters are *m = *0.1; *b* = 0.015; *θ* = 0.5.

In a more realistic case, where deviation from the parents’ foraging behaviour is allowed and there is a possibility for offspring to have different life history traits from their parents, persistence of species in an ecosystem with an unlimited carrying capacity can be possible. In particular, if the saturation parameter *b* in the functional response of the predator is small enough and *D* is sufficiently large, the total biomass of each species shows damped oscillations and approaches a stable stationary state (result not shown here for brevity). At equilibrium, the final distribution of individuals across the cohorts is stationary and its actual shape depends on the parameterization of the trade-off relation *a*(*R*). In Fig. 3 we show the distribution density *ρ_Z_* (*R*) of individuals within the population of zooplankton at equilibrium which is asymptotically stable for both parameterizations of *a*(*R*) (6)–(7) and for different values of *D*. The distribution density *ρ_Z_* (*R*) is defined here as *ρ_Z_*(*R*) = *z*(*R*)/Z, thus integration of *ρ_Z_* (*R*) over all the cohorts gives 1. Fig. 3A shows the equilibrium distribution for the case of linear trade-off function *a*(*R*), whereas Fig. 3B gives equilibrium life history trait distribution for the unimodal parameterizations of *a*(*R*) given by (7).

One can see from Fig. 3 that for large values of *D* the trait distribution of the zooplankton population follows the inverse of the trade-off function *a*(*R*), i.e. 1/*a*(*R*), and this fact can be proven analytically (see Material S2). This signifies that in the case where new born individuals have the same probability of having a given growth rate *R*, regardless of that of their parents, the most abundant cohorts will be those with the smallest vulnerability to predation. Interestingly, for smaller values of *D*, the distribution of individuals *ρ_Z_*(*R*) can be qualitatively different from 1/*a*(*R*). Indeed, one can see from Fig. 3B that monotonically increasing *ρ_Z_*(*R*) can be observed for a non-monotonous function *a*(*R*) exhibiting the minimum vulnerability at intermediate growth rates *R* (see curves 2,3 in Fig. 3B). Note that a qualitatively similar distribution of *ρ_Z_*(*R*) is given by a linear decreasing trade-off function *a*(*R*) (see curve 1 in Fig. 3A). Another important observation from Fig. 3 is that stabilization and persistence of the structured population is possible for various trade-off relations *a*(*R*), showing positive and negative correlation with *R*.

**Figure 4:**
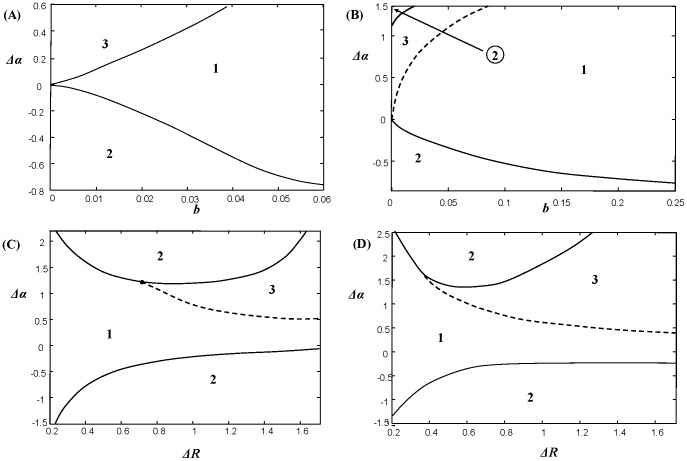
Bifurcation diagrams for predator-prey system (1)–(2) for the linear trade-off relation *a*(*R*) given by (6). Successful top-down control of the system is possible in domains 2 and 3 (the solutions are bounded) and impossible in domain 1. The meaning of the domains is explained in text. Diagrams (A), (B) are constructed for a large value of *D* (*D* = 5), which signifies that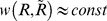; diagrams (C) and (D) are obtained for a smaller *D* (*D* = 0.3). In diagrams (C),(D) the parameter *ΔR* denotes the range of the growth rates *ΔR = R_2_–R_1_*; we consider that (*R_2_+R_1_*)/2 = *const* = 0.85 and *b* = 0.015. The other model parameters are *m = *0.1; *θ* = 0.5, *a_0_* = 1.

Stabilization of the system with a structured prey population is observed within a large range of model parameters. We have constructed a set of bifurcation diagrams showing the possibility of successful top-down control for various trade-off functions; some of which are represented in Fig. 4 (obtained for the linear trade-off function (6): those plotted for the unimodal function (7) are given in Material S3). We consider the key control parameters to be the saturation rate in the predation term, *b*, the parameter *Δα*, determining the gradient of the trade-off relation *a*(*R*), and the parameter *ΔR* which is the range of the growth rates of prey: *ΔR = R_2_–R_1_* (for simplicity, we fix the mean value of *R*, i.e. (*R_2_+R_1_*)/2 = *const*). The diagrams are constructed based on direct numerical simulations of model equations (1)–(2): we determined the type of system behaviour after the transient dynamics die out.

In each diagram, domain 1 corresponds to the situation, where the coexistence stationary state is globally unstable: all trajectories starting nearby will exhibit oscillations with unboundedly increasing amplitude which will result in the eventual extinction of all species. In domain 2, the coexistence state is locally stable, thus small perturbations of this state will eventually vanish. This stationary state, however, is not globally stable and for initial species densities located far away from the state, the trajectories will go to infinity and other factors, such as lack of resources or competition, should limit the population growth in the model. Finally, in domain 3, trajectories unwind from the unstable stationary state and the system exhibits sustained predator-prey oscillations. This limit cycle is not globally stable: large initial deviations of species densities will result in an ecosystem collapse.

One can see from the diagrams that persistence and stabilization in the system requires a supercritical gradient of *a*(*R*). Small variation *ΔR* in the growth rate of individuals should be compensated by a large variation in the vulnerability to predation (large |*Δα*|). In particular, we found that disparity in the growth rates of prey with a constant vulnerability *a* is not enough to ensure system stabilization and species persistence. Also, increasing saturation in the predation *b* will impede the persistence of species in the system: the largest degree of stability is observed for *b* = 0, i.e. in the absence of saturation. The influence of the parameter *D* on the persistence and stability of interactions is not straightforward. We found that an increase of *D* generally increases the area of the domains of persistence, 2 and 3, but can also destabilize the system (cf. Fig. 4C and Fig. 4D). Note that for very small values of *D*, persistence of all species becomes impossible.

**Figure 5:**
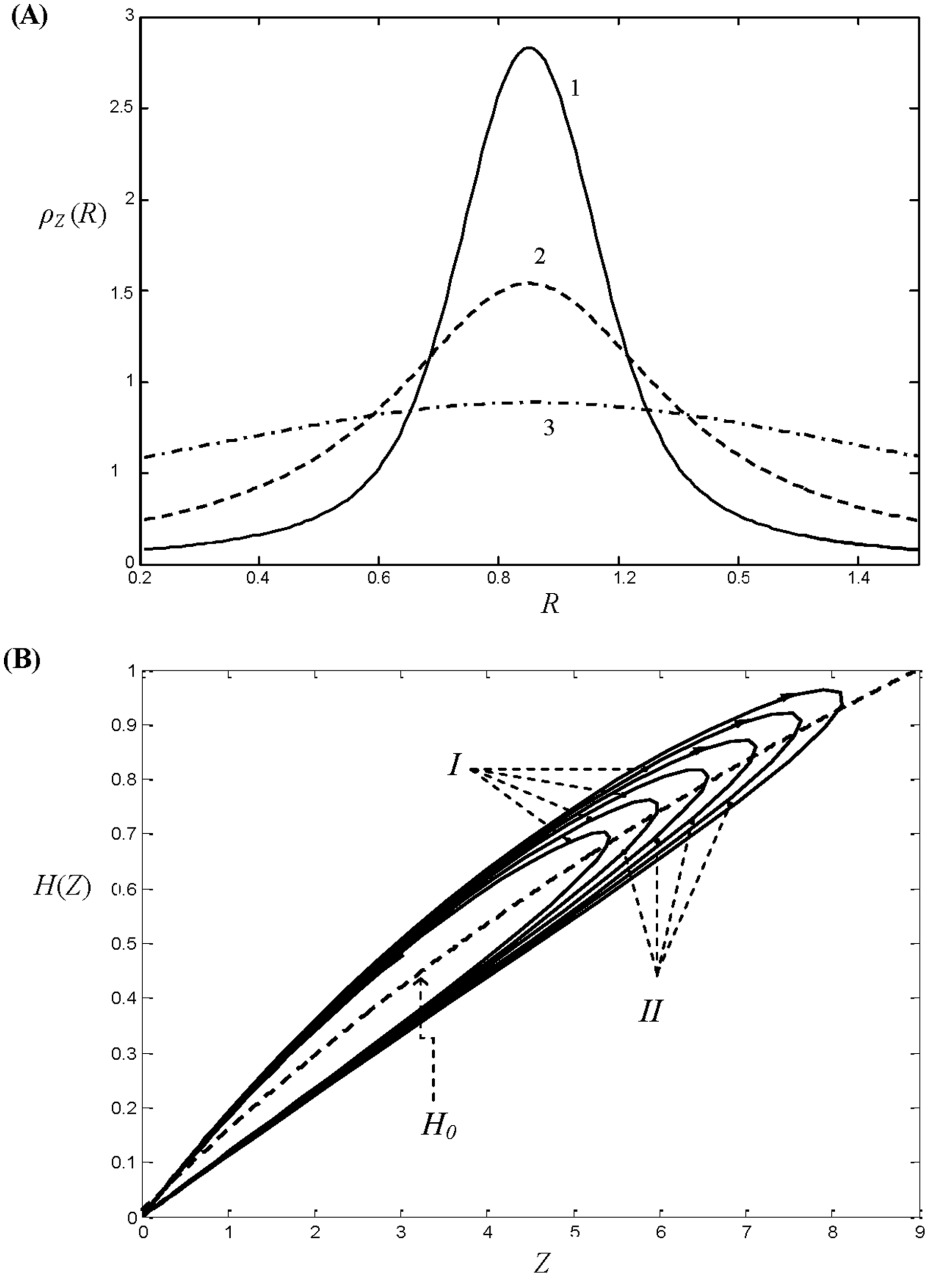
Revealing the mechanism of stabilization in the predator-prey model (1)–(2). The trade-off function *a*(*R*) is given by (8) with *a_0_* = 1; *Δα = *−2. (A) Evolution of the life history trait distribution for the prey population though a cycle. Curves 1 and 3 correspond to the minimal and the maximal predator density though a cycle; whereas curve 2 shows *ρ_Z_* (*R*) for an intermediate density of predator. An increase of predator biomass results in a prevalence of prey cohorts with low vulnerability to predation. (B) The overall functional response of the predator as a function of the total biomass of prey *Z* constructed along system trajectories. The functional response *H_0_* for the equilibrium life history trait distribution *z*(*R*) is shown by dashed line. The direction of motion is shown by arrows. The model parameters are *m = *0.1; *b* = 0.045; *θ* = 0.5.

The crucial question is: what is the mechanism which allows for persistence in the structured population of prey, while it is impossible in the ecosystem where all individuals have the same life history traits? It is possible to come up with a simple (but not mathematically strict) explanation by considering the variation in the distribution of the trait *R* along a predator-prey cycle, both for damped and sustained oscillations. Consider, for instance *a*(*R*) given by (7) with *Δα*<0. In the case, where the density of the predator is near its maximum through the population cycle, the predation term is large compared to the growth rate and this results in selective consumption of prey individuals from more vulnerable cohorts, having large values of *a*, thus the most abundant become individuals with less vulnerability to predation (see Figure 5A, curve 1). When the density of the predator is near its minimum the predation rate becomes smaller compared to the growth term, thus the prey population can recover. The relative proportion of cohorts with small vulnerability to predation is now less pronounced and the distribution of individuals across the cohorts become more even (see Figure 5A, curve 3).

The alteration in the distribution of *R* within the prey population along the cycle affects the predation pressure. This can be seen by plotting the overall functional response *H* of the predator, which gives the overall food intake rate per predator biomass (see Fig. 5B). The overall functional response is computed directly based on the values *z*(*R*) and *F* which evolve in the course of time as long as the trajectory approaches a stable stationary state. For each loop (corresponding to a single predator-prey cycle) we can approximately split *H* into two branches: the upper one, denoted by *I*, corresponds to the ascending part of total prey biomass, the lower one, denoted by *II*, corresponds to the descending of *Z* in the cycle. We have also included the overall functional response of the predator for the equilibrium state of prey population shown by the dashed curve *H_0_*. When *Z* increases, the trophic pressure by *F* is high since *H*>*H_0_* due to the presence of large amount of vulnerable prey individuals. As a result, the prey growth becomes more restricted and the maximal value of Z achieved through the cycle will be smaller compared to the case -where the functional response is fixed and equal to *H_0_*. On the contrary, for the part of the trajectory where *Z* is decreasing, the trophic pressure by *F* is less pronounced (*H*<*H_0_*) and this would result in the minimal value of Z being smaller than in the case where the functional response is *H_0_*. For these reasons, the oscillations of the prey density become damped compared to the case of a fixed functional response *H_0_*, which will eventually result in stabilization of the system provided the saturation coefficient *b* is small enough.

## Discussion

Animal populations are often characterized by a large degree of variability of individuals with respect to behavioural aspects or physiological life traits, even in the case where the organisms in question belong to the same developmental stage and have close biomass. Here we experimentally demonstrate pronounced individual variability in the feeding rates of three herbivorous zooplankton species (see section 2). Our experimental findings inspired us to suggest a parsimonious model to investigate the role of such inter-individual variability in population persistence and stability.

Our main conclusion from the model investigation is that structuring of a population according to some physiological traits and/or individual behaviour can enhance the persistence of the whole population and facilitate a top-down control: predator-prey cycles can be damped and even suppressed. Such a mechanism of stabilization has been missed in previous models where population structuring was considered to be due only to the differences in age and/or body size. The role of variable predation in structured populations has also been somewhat neglected [4], [7], [8], [10], [11]. Unlike previous models, we consider interaction between the zooplankton and their predators in a eutrophic ecosystem with a high level of phytoplankton, thus we disregard the competition between different cohorts of grazers. Note that such competition was a crucial factor in triggering population oscillations and was one of the main focuses of many previous studies (see [11] and the references therein). However, the main difference in terms of model properties is that in an age-structured model a large amount of juveniles will eventually result in the appearance of a pronounced amount of adults, whereas in our model the transition between faster and slower growing cohorts or between more and less vulnerable cohorts is much less straightforward.

The mechanism of top-down control of the structured prey population in model (1)–(2) can be easily understood. At high concentrations of predators, the presence of a large portion of individuals which are less vulnerable to predation within a prey population reduces trophic pressure on the population as a whole. The dominance of less vulnerable cohorts can be considered as a sort of a lifeboat for the whole population during periods of high abundance of the predator. As a result, the prey population will be protected from over-exploitation during the phase of the predator-prey cycle where prey density decreases but the predator density is still high. On the other hand, at low predator densities the distribution of the cohorts becomes more even due to the fact that, in such cases, predation is less important than the growth rate terms in equation (1) for the prey. Also the surviving less vulnerable cohorts can produce individuals belonging to more vulnerable cohorts (due to “mutation”), thus replenishing them. This mechanism is similar to the one reported in [41],[42], where the paradox of the plankton was explained by the existence of eatable and less eatable phytoplankton groups. The difference between this paper and the cited works is that each phytoplankton group in [42] could produce offspring belonging only to the same algal group. In our model the overall functional response of the predator increases (compared to the functional response of a non-structured population) during the phase of the cycle, where the prey population increases, and this prevents the structured prey population from attaining high values, thus dampening the amplitude of oscillations. Surprisingly, successful top-down regulation takes place even in the case of an unlimited carrying capacity for prey and a destabilizing functional response of Holling type II. Note that in earlier models it was reported that intra-population variability would *destabilize* otherwise stable dynamics [10], [11], [43], with the main destabilizing factor supposedly being competition for food between the juveniles and adults.

The key-issue guaranteeing top-down control in model (1)–(2) was the assumption about the existence of a certain trade-off relation between the growth rate of the zooplankton *R*, and their vulnerability to predation, *a*. As basic examples, we have considered the linear and parabolic parameterizations (6)–(7), thus representing monotonic and unimodal dependences *a*(*R*). The most surprising observation is that the actual shape of the trade-off function *a*(*R*) does not play a major role in the stabilization of population cycles. Indeed, as it follows from the diagrams in Fig. 4, the main condition for regulation in the model is not the particular shape of *a*(*R*) but the magnitude of the gradient of *a*(*R*) (see also the Material S3). We found that similar top-down control is possible for more sophisticated parameterizations of *a*(*R*), for instance, for a function having several maxima and minima (result not shown here for brevity), provided the gradients of *a*(*R*) within some ranges of *R* are supercritical. Note that in the case where the inter-individual variability involves only the growth rate, this will be insufficient to ensure stabilization and population persistence. On the other hand, we found that variation of *a* alone with a constant value of *R* also cannot ensure successful top-down control: we need unevenness both in the growth rate and in the vulnerability to predation across the cohorts and the degree of variation needs to be sufficiently large.

We should emphasize that the stabilization in model (1)–(2) requires that the life history traits and behavioural patterns of individuals remain constant though their life span; in other words, the organisms should not change their cohorts. We shall refer to such structuring as long term structuring or genetic structuring of a population. In contrast, there can also be short term (temporal) structuring within a population where the life history traits of each cohort randomly vary in time. As a result, the splitting of a population into cohorts occurs for a short time period (compared to the individual lifespan) after which the individuals swap between different cohorts. A typical example of temporal structuring of a population is the influence of the feeding history of individuals, where organisms within a population are exposed to different food densities and can have different fecundity at a given moment of time. This case is usually described by population models with delay [11], [43] for which destabilization of the stationary state would occur for a critical value of delay. In model (1)–(2) temporal structuring can also result in destabilization in the case that a fast random transition between the cohorts is allowed.

We should emphasize that in reality physiologically structured populations neither exhibit pure genetic structuring nor pure temporal structuring: organisms can eventually swap cohorts; however this can be a rare event [5], [6]. For instance, this can explain the fact that in our feeding experiments (Fig. 2) we do not have the correlation coefficient in Fig. 2 close to 1. We can incorporate such a scenario by introducing a term into equation (1) allowing for exchange between the cohorts. In the simplest case we can assume that the exchange between cohorts is proportional to the number of individuals and that it takes place between the nearest cohorts; thus we can describe the process in terms of a classical diffusion. Thus, equation (1) becomes
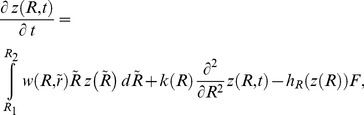
(8)where the coefficient *k*(*R*) is the a diffusion coefficient, which can be also a function of *R*. This term describes transition of individuals across the cohorts during the life span and does not model the partitioning of offspring.

We briefly considered possible consequences of transitions between cohorts described by (8) for a constant *k*, and we found that stabilization in the system is possible for small *k* (e.g. *k*≡0.5), but for large *k* it becomes seriously hampered within the considered range of parameters (e.g. for *k*≡5 it is impossible). Indeed, in this situation at each moment of time the distribution of organisms according to their life history traits distribution is random. Assuming that this distribution is described by the Gaussian law, integration over the cohorts will give a standard predator-prey model for the total population sizes of species which is globally unstable (with an unlimited carrying capacity) [40].

Empirical confirmation of the stabilization mechanism found in model (1)–(2) would be an important extension of this work and should include an accurate comparison of the distribution of life history traits in the population of grazers at different levels of predation load in the system. Some insights, however, can be obtained from the feeding experiments described in section 2 of this paper. In particular, in the experiments the distribution of the ingestion rates of some species (e.g. *C. finmarchicus*) cannot be considered as a classical Gaussian law (see Fig. 1A) emerging due to a random distribution around a certain mean value. Thus, we can suggest that the current distribution of the population of grazers directly collected in the ocean is not only a result of random variation of life history traits, but is possibly also due to trophic interaction with predators which have been selectively consuming the most vulnerable grazers. On the other hand, the unimodal distribution in the other two cases (Fig. 1B,C), which can be approximated by the normal distribution, may in reality have a different origin as an interplay of a large number of independent factors resulting in a Gaussian distribution. Indeed, in model (1)–(2) we found that *a*(*R*) given by (6) with *Δα*<0 for the distribution of individuals across the cohorts may be quite close to the normal distribution (although, mathematically it has a different formulation) and the implementation of standard statistical packages (e.g. Microsoft EXCEL) might indicate the ‘normality’ of *ρ_Z_* (*R*). In this case, variability of individuals within a population can be wrongly concluded to be a result of only random factors, whereas in reality the life history traits distribution is largely shaped by deterministic factors such as selective grazing by predators.

The enhancement of persistence and stabilization obtained in the basic model (1)–(2) is quite robust with respect to certain modifications which can be done to make the model more realistic. In particular, when describing the effects of saturation in the functional response (5), we assumed that the half-saturation density 1/*b* was the same for all cohorts. However, if we assume that the vulnerability to predation is a function of time spent in the surface layer [44], [45], the actual amount of zooplankton which is available for predation by visual predators should be given by the sum of the density of cohorts multiplied by certain weights. Those weights would model the relative duration of the zooplankton cohorts’ stays in the more risky environment, thus they should be a function of the predator attack rate. We should say that including this constraint does not affect the main results, provided the saturation rate 1/*b* is high enough. Another modification of (1)–(2) is to take into account competition between the zooplankton cohorts for a common resource (phytoplankton), in which case the growth rate *R* will decrease for large values of *Z*. In the simplest case, this can be done by adding a carrying capacity *K* for the prey population in equation (1). We found that in the case of a finite carrying capacity (e.g. *K* = 200), successful top-down control of the system is possible in a much broader region of the parameter space.

Since our results are obtained based on a rather generic model, they can be applied to some other non-planktonic predator-prey systems. In particular, pronounced differentiation of individuals according to their life history traits and/or behaviour has been found in fish [2], [5], [15], [46], octopuses [13] and some mammals [16]. In particular, it was reported in a population of salmon that the inter-individual variability in terms of willingness to take predation risk near the surface could result in structuring of the patterns of vertical migration behaviour in the water column [14]. Thus, structuring in those populations could provide an extra degree of stability and enhance persistence of those species. Finally, we emphasize that this study should not be interpreted as denying the importance of age and/or size structuring in population dynamics, in particular when modelling interactions between herbivorous zooplankton and their predators. It is well known that different developmental stages of copepods show distinctly different food intake rates, [47]–[49] and clearly juveniles cannot produce offspring as was the case model (1)–(2). The same suggestion concerns the population of predators: carnivorous zooplankton and planktivorous fish. Thus it would be interesting to combine both the present modelling approach considering physiologically-based structuring, and the basic age/sized structured population approach. We expect that in this case a crucial factor will be the level of resources available for the structured population of grazers, and such a combined approach should allow us to estimate which mechanism will eventually prevail with various resource levels: destabilization due to competition between juveniles and adults for resources (i.e. an indirect bottom-up control through intra-population competition) or stabilization due to different consumption by predators throughout the population cycle (i.e. top-down control). We are planning to address this issue in detail in future studies.

## Supporting Information

Material S1 **Experimental estimation of grazing rates of herbivorous copepods.**
(PDF)

Material S2 **Analytical investigation of the properties of the stationary state of model (1)–(2).**
(PDF)

Material S3 **Supplementary bifurcation diagram for model (1)–(2).**
(PDF)
