# Supplementary material for: Revisiting the Role of Individual Variability in Population Persistence and Stability
Source: PLoS One. 2013 Aug 2;8(8):e70576. doi: 10.1371/journal.pone.0070576 (PMC3732237; doi:10.1371/journal.pone.0070576)
Supplement: Material S1 — Experimental estimation of grazing rates of herbivorous copepods. (PDF) [file pone.0070576.s001.pdf]

Supplementary Material S1.

(*Morozov, Pasternak and Arashkevich*)

Experimental estimation of grazing rates of herbivorous copepods

For each species, organisms were sampled in the upper 200 m with WP-2 plankton net (200 -μm mesh) and gently poured into large vessels with filtered sea water. In the laboratory, healthy, undamaged copepods were immediately collected and placed individually into vertically positioned 50-ml wide mouthed tubes. The tubes were filled with a feeding suspension (345 cells ml^{-1} of the diatom *Thalassiosira gravida* that corresponds to 160 μg C l^{-1}). The concentrations were chosen to reflect those which occur during the periods of phytoplankton bloom (Smayda, 1997; Moncheva et al., 2001). The concentration of phytoplankton cells was estimated prior to each experiment under an inverted microscope. Copepods were allowed to adapt to experimental conditions for 24 hrs. The following day, copepods were transferred into the fresh medium under the same conditions and allowed to feed during 3 hours for *C. glacialis*, 4 hours for *C. finmarchicus* and 5 hours for *C. euxinus*. After completion of feeding experiments, copepods were transferred into a fresh medium and left feeding until the next trial (the following day). The chosen duration of each trial was the longest for the species with the smallest body size (*C euxinus*) and shortest with the largest (*C. glacialis*) to ensure similarly reliable estimations of feeding rate.

The ingestion rate of *C. glacialis* and *C. finmarchicus* was calculated considering the difference between the concentration of Chl *a* before and after feeding (measured based on the fluorescence method) according to the following equation:

$$I = (C_0 - C_t) / \tau,$$

where *I* is the ingestion rate, C_0 and C_t are the food concentrations at the beginning and the end of experiment, respectively, and τ is the incubation time (for detail see ICES Zooplankton Methodology Manual, 2000). Chl *a* was used as a proxy for phytoplankton concentration. Pigment concentration was calculated by the standard fluorometric procedure, with reading before and after acidification with 10% HCl (Parsons et al., 1984). To avoid eventual underestimation of the ingestion rate, egested faecal pellets were thoroughly collected and rinsed, pellets taken away and the rinsing water added to the suspension. Further, 50 ml of the initial suspension (3 replicates) and the total volume left after each individual feeding session were filtered onto a GF/F filter and extracted in pure methanol for 24 hrs in the dark (Huntley et al. 1987; Pasternak et al., 2008)

Estimation of ingestion rates of *Calanus euxinus* was based on another approach. We counted and measured the length and width of the faecal pellets egested during each feeding session. The total volume of the pellets egested by each specimen during a single trial was calculated, assuming the shape of the pellets is close to that of a cylinder. In our investigation we use the fact that egested faecal material has been shown to be a reliable proxy for feeding rate (Ayukai and Nishizawa, 1986; Tsuda and Nemoto, 1990; Pasternak et al., 2004). We used this approach to compare the individual feeding activity in the third experiment (*C. euxinus*), as this simple technique allowed us to increase the number of replicates.

References

Ayukai, T., and S. Nishizawa. 1986. Defecation rate as a possible measure of ingestion rate of Calanus pacificus (Copepoda: Calanoida). Bulletin of Plankton Society of. Japan, 33: 3-10.

Huntley, M., K. Tande, and H.C., Eilertsen. 1987. On the trophic fate of Phaeocystis pouchettii (Hariot). II. Grazing rates of Calanus hyperboreus (Kroyer) on diatoms and different size

categories of Phaeocystis pouchettii. Journal of Experimental Marine Biology and Ecology 110: 197–212.

ICES Zooplankton Methodology Manual. 2000. Eds.: Harris R.P., Wiebe P.H., Lenz J., Skjodal H.R., Huntley M. Academic Press, London, 684 pp

Moncheva S., O. Gotsis-Skretas, K. Pagou, and A. Krastev. 2001. Phytoplankton Blooms in Black Sea and Mediterranean coastal ecosystems subjected to anthropogenic eutrophication: Similarities and differences. Estuarine, Coastal and Shelf Science 53: 281–295.

Parsons, T.R., Y. Maita, and C.M. Lalli.1984. A manual of chemical and biologicqal methods for seawater analysis. Pergamon Press, Oxford, 187 pp.

Pasternak A., K.S., Tande E. Arashkevich, and W. Melle. 2004. Reproductive patterns of Calanus finmarchicus at the Norwegian midshelf in 1997. Journal of Plankton Research, 26: 839-849.

Smayda, T.J. 1997. What is a bloom? Limnology and Oceanography 45: 1132-1136.

Tsuda, A., and T. Nemoto. 1990. The effect of food concentration on the faecal pellet size of the marine copepod Pseudocalanus newmani Frost. Bulletin of Plankton Society of Japan 37: 83-90.
