# Supplementary material for: Revisiting the Role of Individual Variability in Population Persistence and Stability
Source: PLoS One. 2013 Aug 2;8(8):e70576. doi: 10.1371/journal.pone.0070576 (PMC3732237; doi:10.1371/journal.pone.0070576)
Supplement: Material S2 — Analytical investigation of the properties of the stationary state of model (1)–(2). (PDF) [file pone.0070576.s002.pdf]

Supplementary Material S2

(*Morozov, Pasternak and Arashkevich*)

Here we analytically investigate the properties of the stationary state of model (1)-(2) which are the solutions for the following equations:

$$\int_{R_1}^{R_2} w\left(R,\tilde{R}\right)\tilde{R}z\left(\tilde{R}\right)d\tilde{R} - a(R)\frac{z(R)F}{1+bZ} = 0\,, \qquad \text{(B1)}$$

$$\theta\int_{R_1}^{R_2} \frac{a(R)z(R)}{1+bZ}dR - m = 0\,. \qquad \text{(B2)}$$

Note that solving the system of integral equations with the demographic kernel given by (4) with an arbitrary *D* is a rather complicated issue and it should be addressed in detail elsewhere. Here we consider a particular case where *D* is sufficiently large and we can assume that $w\left(R,\tilde{R}\right) = const = 1/\left(R_2 - R_1\right)$, thus the growth rate *R* is distributed uniformly among the offspring of all cohorts. From (A1) we have

$$z(R) = \frac{1+bZ}{(R_2 - R_1)a(R)F}\int_{R_1}^{R_2} R\,z(R)dR\,, \qquad \text{(B3)}$$

where Z is the total biomass of zooplankton.

Expression (B3) gives the stationary shape of $z(R)$, i.e. the *p*-state of the population of prey. One can see that it is inversely proportional to the vulnerability to predation *a*(*R*). We further substitute $z(R)$ into (B2)

$$\theta\int_{R_1}^{R_2} a(R)\frac{dR}{a(R)F}\int_{R_1}^{R_2} R\,z(R)d\,R = m \qquad \text{(B4)}$$

It can be easily derived that

$$F = \frac{\theta}{m} \int_{R_1}^{R_2} R\, z(R)\, dR \qquad \text{(B5)}$$

We substitute (B5) into (B3) and obtain

$$z(R) = m \frac{1 + bZ}{(R_2 - R_1)a(R)\theta}, \qquad \text{(B6)}$$

and by integrating both sides of (A6) we get

$$Z = \frac{m}{\theta} \frac{(1 + bZ)}{(R_2 - R_1)} \int_{R_1}^{R_2} \frac{dR}{a(R)}, \qquad \text{(B7)}$$

which gives

$$Z = \frac{\dfrac{m}{\theta} \displaystyle\int_{R_1}^{R_2} \frac{dR}{a(R)}}{1 - b\dfrac{m}{\theta} \displaystyle\int_{R_1}^{R_2} \frac{dR}{a(R)}}. \qquad \text{(B8)}$$

The expression for the *p*-state of the population of prey is given by (B6) where the total biomass of prey can be computed from (B8). The total biomass of predator can be determined from (B5) by integrating the explicit expression for $z(R)$.
