# Supplementary material for: Revisiting the Role of Individual Variability in Population Persistence and Stability
Source: PLoS One. 2013 Aug 2;8(8):e70576. doi: 10.1371/journal.pone.0070576 (PMC3732237; doi:10.1371/journal.pone.0070576)
Supplement: Material S3 — Supplementary bifurcation diagram for model (1)–(2). (PDF) [file pone.0070576.s003.pdf]

Supplementary Material S3

(*Morozov, Pasternak and Arashkevich*)

Here we present a supplementary bifurcation diagram (see Figure S3) for model (1)-(2) constructed numerically in the case where the trade-off relation relation $a(R)$ is not monotonic and given by the parabolic parameterization (7). Successful top-down control of the system is possible in domains 2 and 3 (the solutions are bounded) and impossible in domain 1. The meaning of the domains is explained in the main text. Diagrams (A), (B) are constructed for a large value of D (D=5), which signifies that $w(R, \tilde{R}) \approx const$; diagrams (C) and (D) are obtained for a smaller D (D=0.3). In diagrams (C), (D) the parameter ΔR denotes the range of the extreme growth rates $\Delta R=R_2–R_1$; we consider that $(R_2+R_1)/2=const=0.85$ and b=0.015.The other model parameters are m=0.1; θ=0.5, a_0=1.

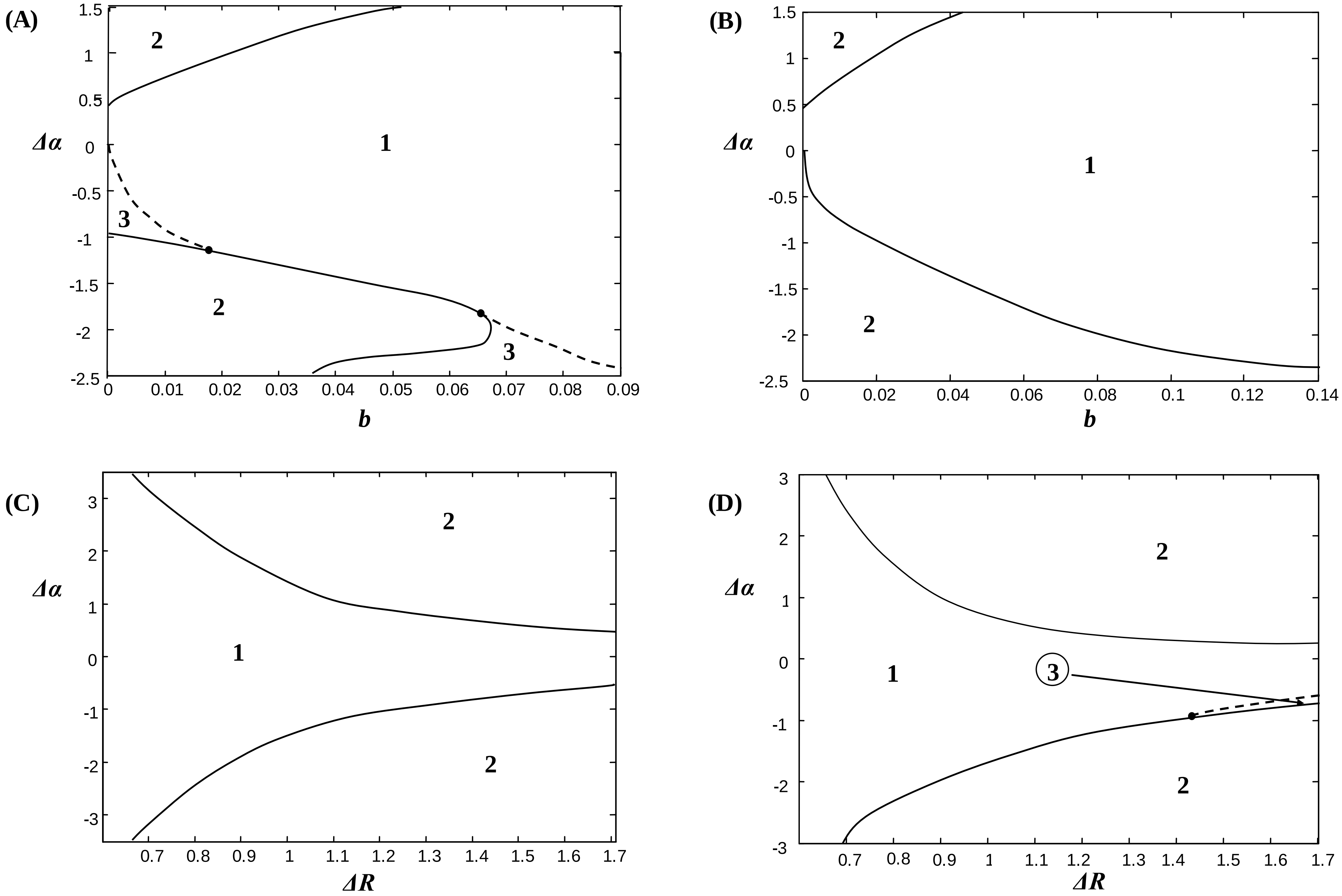

Figure S3
